# Evaluation of Severe Acute Respiratory Syndrome surveillance caused by respiratory viruses in a pediatric unit, 2013 to 2019

**DOI:** 10.1590/1984-0462/2024/42/2022215

**Published:** 2023-08-25

**Authors:** Felipe Teixeira de Mello Freitas, Cíntia Costa Pereira Pimentel, Pedro Ribeiro Bianchini, Rafaela Moreira de Carvalho, Alexandre Peixoto Serafim, Cira Ferreira Antunes Costa

**Affiliations:** a Escola Superior de Ciências da Saúde, Brasília, DF, Brazil.; b Hospital Materno Infantil de Brasília, Brasília, DF, Brazil.

**Keywords:** Severe acute respiratory syndrome, Respiratory syncytial virus, Influenza, Program evaluation, Epidemiological surveillance services, Brazil, Síndrome respiratória aguda grave, Vírus sincicial respiratório, Influenza, Avaliação de programas e projetos de saúde, Serviços de vigilância epidemiológica, Brasil

## Abstract

**Objective:**

To evaluate severe acute respiratory syndrome surveillance in a pediatric unit.

**Methods:**

Descriptive study of reported severe acute respiratory syndrome cases with the detection of respiratory viruses in the nasopharyngeal sample of patients hospitalized between 2013 and 2019, in a reference hospital in the Federal District, Brazil.

**Results:**

A total of 269 children had one or more viruses detected, resulting in 280 viruses, of which 152 (54%) were respiratory syncytial virus. The detection of respiratory syncytial virus was higher during the autumn-winter period. Children´s median age was 6.9 months, 156 (58%) were male, 104 (39%) had comorbidity, 197 (73%) required mechanical ventilation, 241 (90%) received antibiotics, and 146 (54%) oseltamivir. There were 19 (7%) deaths. The median time from symptom onset to sample collection was 5 days and the median time from sample collection to final results was 6 days.

**Conclusions:**

The system needs to reduce the time to deliver results so that inappropriate use of antibiotics and antivirals can be avoided. Moreover, the burden of viral pneumonia was relevant and the system must be flexible enough to include emerging viruses in order to be useful in responding to public health emergencies caused by respiratory viruses.

## RESUMO

**Objetivo:**

Avaliar a vigilância da síndrome respiratória aguda grave em uma unidade pediátrica.

**Métodos:**

Estudo descritivo dos casos de síndrome respiratória aguda grave, notificados e com a detecção de vírus respiratório em amostra de nasofaringe de pacientes internados entre 2013 e 2019, em um hospital de referência do Distrito Federal.

**Resultados:**

Um total de 269 crianças tiveram algum vírus detectado, resultando em 280 vírus, sendo 152 (54%) vírus sincicial respiratório. A detecção do vírus sincicial respiratório foi maior durante o período de outono-inverno. A mediana da idade das crianças foi de 6,9 meses, 156 (58%) eram do sexo masculino, 104 (39%) tinham comorbidade, 197 (73%) necessitaram de ventilação mecânica, 241 (90%) receberam antibióticos e 146 (54%) oseltamivir. Ocorreram 19 (7%) óbitos. A mediana do tempo desde o início dos sintomas até a coleta da amostra foi de 5 dias, e do tempo da coleta até o resultado foi de 6 dias.

**Conclusões:**

O sistema necessita reduzir o tempo do resultado final para que seja possível evitar o uso inadequado de antibióticos e antivirais. Ademais, o impacto das pneumonias virais foi relevante e o sistema deve ser flexível suficiente para incluir vírus emergentes, para ser útil na resposta às emergências de saúde pública causada por vírus respiratórios.

## INTRODUCTION

In recent years, the increased ability to diagnose airway infections by molecular biology has expanded our knowledge of the role and circulation of respiratory viruses.^1^ In a population-based active surveillance study, conducted from 2010 to 2012 in the United States, among children hospitalized for pneumonia, the etiological agent identified was virus in 66% of cases and viral and bacterial coinfection in 7% of cases; the main agent detected was respiratory syncytial virus (RSV), followed by adenovirus and metapneumovirus, especially in children under 5 years old.^2^ Similar results regarding the etiology of pneumonia in children were observed in a similar study carried out in Ecuador between 2008 and 2010.^3^ In Brazil, RSV was also the main agent identified in children under 15 years of age by the national sentinel surveillance of influenza and other respiratory viruses.^4^ Lower respiratory tract infections due to RSV was estimated to cause 3,2 million hospitalizations and 60,000 deaths per year worldwide in children under 5 years old.^5^

In Brazil, influenza and other respiratory viruses surveillance was implemented in the year 2000, based on sentinel emergency care units aiming to identify circulating viruses, monitor trends in care for influenza-like illness (ILI), evaluate the impact of influenza vaccination, and respond to unusual situations.^6^ As of 2009, due to the influenza A H1N1 pandemic, the surveillance system began to include universal notification and collection of material from the nasopharynx to investigate respiratory viruses in hospitalized patients who met the criteria for severe acute respiratory syndrome (SARS), that is, individuals with signs and symptoms of ILI who had dyspnea or other signs of severity.^7^ The collected samples were sent to the Central Public Health Laboratories (*Laboratório Central de Saúde Pública -* LACEN) of each federated unit, which were responsible for identifying the etiologic agent, typing, and subtyping of circulating influenza viruses. Then, a quantity of these samples was sent to the three national reference laboratories, responsible for the antigenic and genetic characterization of circulating viruses, identification of new subtypes, testing for resistance to antivirals, and sending viral isolates to the collaborating center of the World Organization for Health (WHO) of the Americas to support the selection of viral strains for the annual composition of the influenza vaccine.^8,9^

Surveillance was primarily aimed at the adult population with a focus on influenza viruses, but pediatric units were allowed to participate. The Hospital Materno Infantil de Brasília (HMIB), located in the Federal District, Brazil, started reporting SARS cases and collecting material for viral identification in 2012. The objective of the present study was to evaluate different aspects of respiratory viruses surveillance in a pediatric unit prior to the COVID-19 pandemic, including the viral agents identified and their circulation during the year, patients´ demographics, clinical outcomes, antibiotic and antiviral prescribing practices, and the surveillance system’s ability to provide a timely response.

## METHOD

This is a descriptive study of cases of viral pneumonia, which met SARS criteria, confirmed by the detection of respiratory viruses in a nasopharyngeal sample, admitted to the emergency room (ER) or pediatric intensive care unit (ICU) of the HMIB from January 2013 to December 2019. The HMIB is a reference pediatric hospital but with an ER open to free demand, which receives patients from the Federal District area and surroundings. The HMIB’s ICU has 12 beds, 2 of which have isolation capacity, and receives patients from one month to 14 years old, regulated from the entire Federal District and neighboring municipalities.

All children admitted to the HMIB who met the SARS criteria and had a virus detected in the nasopharyngeal sample collected during the study period were included in the study. The data were obtained from the notifications registered in the National Information System for Notifiable Diseases (*Sistema de Informação Nacional de Agravos de Notificação –* SINAN) — Influenza database from 2013 to 2018 and in the Epidemiological Surveillance Information System (*Sistema de Informação de Vigilância Epidemiológica –* SIVEP) — Influenza of 2019. Additionally, the medical records were reviewed to obtain hospitalization data that were not included in the notification form.

The variables collected were related to the patient (sex, age, date of onset of symptoms, and presence of comorbidities), to the nasopharynx sample (viruses identified, date of sample collection, and date of result) and to the hospital admission (date of hospital and ICU admission, use of mechanical ventilation, prescription of antibiotics or antivirals, and clinical outcome: hospital discharge or death).

The viral etiological diagnosis was performed by the Federal District´s LACEN through the processing of respiratory secretion samples collected by nasopharynx and oropharynx swab. Initially LACEN performed the indirect immunofluorescence (IIF) technique, whose positive samples were confirmed by polymerase chain reaction (PCR) from 2013 to 2015. As of 2016, LACEN started to use only PCR for sample processing. A panel with the following viruses was used: influenza A, influenza B, RSV, adenovirus, parainfluenza 1, parainfluenza 2, parainfluenza 3, and metapneumovirus. Rhinovirus and bocavirus were included in 2019.

A descriptive analysis was performed. Discrete variables were categorized and presented through proportions. Continuous variables were presented using the median and interquartile range (IQR).

The study was approved by the Ethics Committee of the Foundation for Teaching and Research in Health Sciences (*Fundação de Ensino e Pesquisa em Ciências da Saúde –* FEPECS) of the Federal District: protocol number CAAE 15812419.3.0000.5553.

## RESULTS

During the study period, HMIB reported 1,387 children admitted as SARS who had a nasopharyngeal sample collected for respiratory virus detection; 269 (19%) children had a viral detection and were included in the study. From 2014 to 2019, a total of 2,176 children were admitted to the ICU of the HMIB and 921 (42%) were diagnosed with respiratory infection. There was a gradual increase in SARS notifications over the years (Table 1).

**Table 1:** Number of pediatric intensive care unit admissions, pediatric intensive care unit admissions due to respiratory conditions, reported cases of severe acute respiratory syndrome, and cases of severe acute respiratory syndrome with virus detection in a pediatric unit, Brasília (DF), 2013 to 2019.

Year	Pediatric ICU admissions	Pediatric ICU admissions due to respiratory conditions n (%)	Reported cases of SARS	Cases of SARS with virus detection n (%)
2013	332	---	38	8 (21)
2014	331	96 (29)	23	4 (17)
2015	259	83 (32)	28	5 (18)
2016	293	122 (42)	91	26 (28)
2017	381	154 (40)	162	43 (26)
2018	407	190 (47)	430	62 (14)
2019	505	276 (55)	615	121 (20)

ICU: intensive care unit; SARS: severe acute respiratory syndrome.

**Figure 1:**
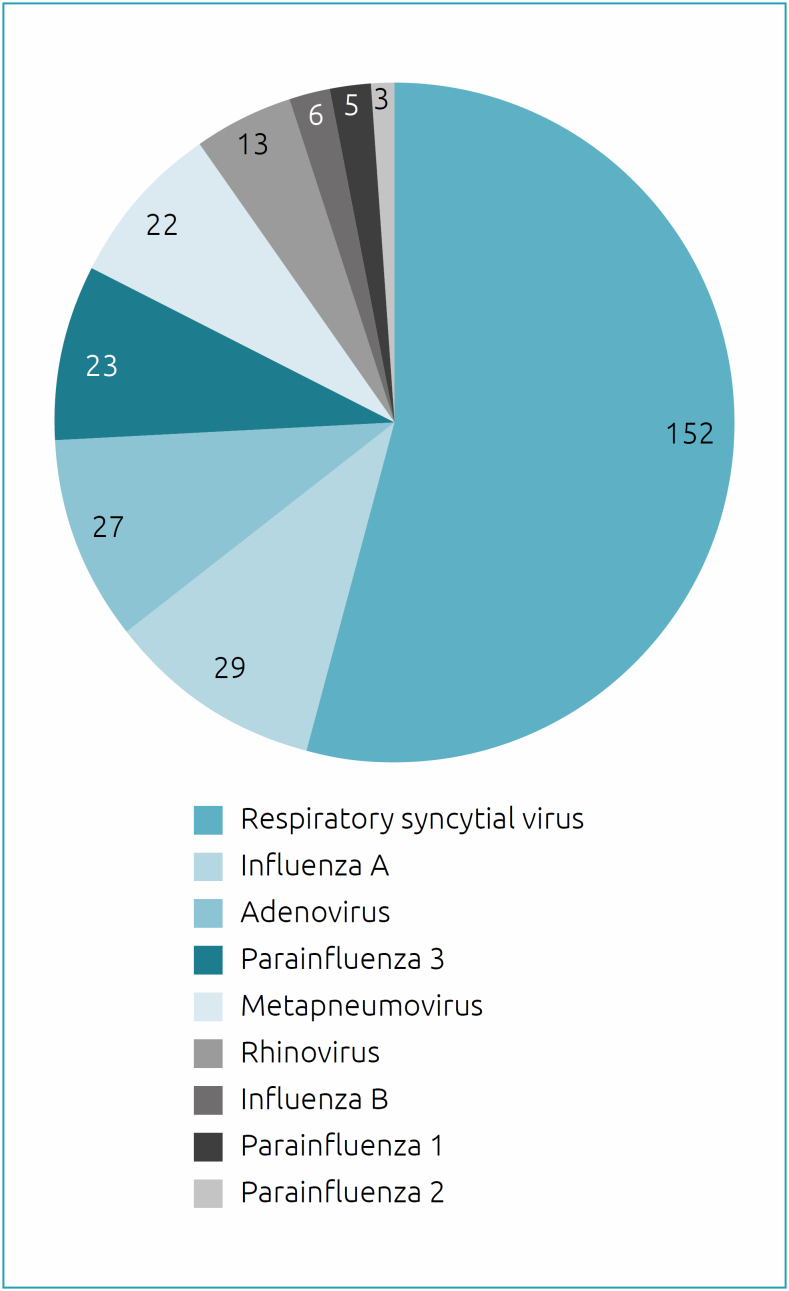
Respiratory viruses detected in a pediatric unit, Brasília (DF), 2013 a 2019.

Of the 269 children, 280 were detected as viruses, 152 were RSV (54%), 29 influenza A (10%), 27 adenovirus (10%), 23 parainfluenza 3 (8%), 22 metapneumovirus (8%), 13 rhinovirus (5%), 6 influenza B (2%), 5 parainfluenza 1 (2%), and 3 parainfluenza 2 (1%) (Figure 1). There were 11 cases of co-infection: 3 of RSV and adenovirus, 2 of RSV and influenza A, 2 of RSV and metapneumovirus, 2 of parainfluenza 2 and adenovirus, 1 of RSV and parainfluenza 3, and 1 of metapneumovirus and adenovirus.

**Figure 2:**
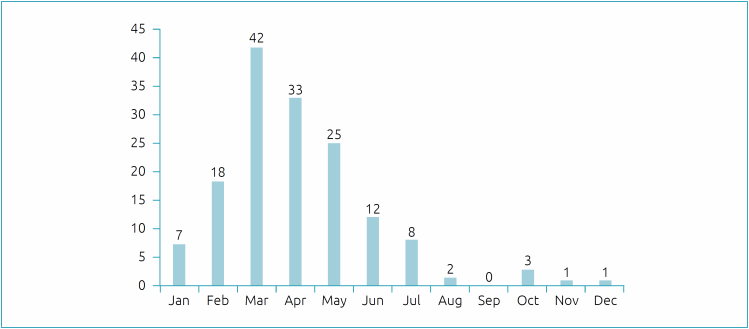
Monthly distribution of cases of respiratory syncytial virus in a pediatric unit, Brasília (DF),2013 a 2019.

The detection of RSV was higher during the months of February to June, corresponding to the autumn-winter period. The seasonality of RSV is shown in Figure 2.

Among the 269 children, 156 (58%) were male, the median age of the cases was 6.9 months (IQR: 2.2–18.3 months), and 104 (39%) had some comorbidity related to increased risk of progression to severe respiratory infection: heart disease, lung disease, prematurity or low birth weight, among others. Thirty-three (12%) had more than one comorbidity. One hundred and ninety-seven (73%) required mechanical ventilation (MV), the median duration of MV was 5 days (IQR: 4–9 days). There were 19 (7%) deaths, of which 11 had comorbid conditions. Table 2 presents the children’s data stratified by the five main viruses detected.

**Table 2:** Data from children hospitalized with severe acute respiratory syndrome stratified by the five main viruses identified in the nasopharyngeal sample in a pediatric unit, Brasília (DF), 2013 to 2019.

	RSV (n=152)	Influenza A (n=29)	Adenovirus (n=27)	Parainfluenza 3 (n=23)	Metapneumovirus (n=22)
Male sex, n (%)	91 (60)	17 (59)	17 (63)	14 (61)	12 (54)
Age in months, median (IQR)	3.5 (1.5–12.7)	15.7 (10.1–49.3)	20.4 (12.6–44.2)	6.0 (3.5–19.5)	9.0 (4.0–13.1)
Comorbidity, n (%)	53 (35)	7 (24)	11 (41)	13 (56)	11 (50)
Mechanical ventilation use, n (%)	115 (76)	22 (76)	22 (81)	14 (61)	14 (64)
Deaths, n (%)	5 (3)	2 (7)	4 (15)	2 (9)	2 (9)

RSV: respiratory syncytial virus; IQR: interquartile range.

Two hundred and forty-one (90%) received some antibiotic at hospital admission: 90 (37%) received the combination of ampicillin and sulbactam, 55 (23%) received ceftriaxone, and 31 (13%) received ampicillin. Oseltamivir was prescribed to 146 children (54%), of which 21 (14%) had the detection of influenza virus. Among the 123 children (46%) who did not receive oseltamivir, 14 (11%) had the detection of influenza virus.

Six (2%) children developed symptoms when they were already hospitalized, 5 in the ward and 1 in the ICU, which configures intra-hospital transmission and cases of healthcare-associated infections. Of the 263 children admitted from the community, the median time between symptom onset and hospital admission was 2 days (IQR: 1–4 days) and the median time between symptom onset and ICU admission was 4 days (IQR: 2–6 days) among 268 children. The median time between the onset of symptoms and the collection of a nasopharyngeal sample was 5 days (IQR: 3–7 days) and between hospital admission and the collection of a nasopharyngeal sample was 2 days (IQR: 1–4 days). The median time between collection of nasopharyngeal material and the result of the viral detection was 6 days (IQR: 4–9 days). The median total length of stay in the ICU and hospital was 6 days (IQR: 3–10 days) and 12 days (IQR: 8–20 days), respectively. A summary with the time interval of the various steps of the surveillance system is presented in Table 3.

**Table 3:** Time interval in days of different steps of viral pneumonia surveillance in a pediatric unit, Brasília (DF), 2013 to 2019.

Time interval in days		Median (IQR)
From symptom onset to hospital admission		2 (1–4)
From symptom onset to ICU admission		4 (2–6)
Length of stay in the ICU		6 (3–10)
Length of stay in the hospital		12 (8–20)
From symptom onset to sample collection		5 (3–7)
From hospital admission to sample collection		2 (1–4)
From sample collection to final results	
	IIF	6.5 (4–9)
	PCR	6 (4–9)

IQR: interquartile range; ICU: intensive care unit; IIF: indirect immunofluorescence; PCR: polymerase chain reaction.

## DISCUSSION

Our study evaluated SARS surveillance in a pediatric unit and demonstrated that the impact of respiratory infections in a pediatric health service was already relevant before the COVID-19 pandemic, with hospitalizations for respiratory infection accounting for up to half of the ICU admissions. This proportion may be even higher during the autumn-winter period, which is the seasonal period of RSV, the main etiological agent that leads to the admission of children to the ICU, especially those under one year of age. In addition, cases of nosocomial transmission of respiratory viruses were also verified, highlighting their role as a cause of healthcare-associated infections, which pose more challenges in their control during epidemic periods.

In fact, RSV was detected in just over half of the samples studied, especially in children under one year of age. RSV infection is the leading cause of lower respiratory tract infection in children worldwide; it is estimated that lower respiratory tract infections due to RSV cause about 3,2 million hospitalizations and about 60,000 deaths per year worldwide in children under 5 years of age, affecting developing countries more severely.^5,10^ In a systematic review of studies carried out in Latin America, it was stated that RSV is the main etiology of lower respiratory tract infection in children up to 11 months of age and has a fatality rate of 1.74%,^11^ similar to our results. Therefore, RSV has been the target of vaccines, which despite advances, there is still no approved and available vaccine against the virus.^12,13^ The only form of prevention currently available is the administration of palivizumab, a monoclonal antibody against RSV, for groups at risk for severe complications of the infection during the seasonal period of virus circulation.^14,15^ In Brazil, between 2008 and 2015, hospitalizations for acute lower respiratory infections in children under one year old showed increasing rates; the incidence of hospitalization decreased one year after palivizumab implementation but increased again in the second year of the program.^16^ Our study found a period of RSV intensity during autumn-winter, from February to July, the same seasonality reported in the national data and which is in accordance with the period of palivizumab application in the Federal District.^4,17^ However, due to the high cost and concerns about the cost-effectiveness of palivizumab, it is only applied to high-risk patients for complications from RSV infection, with some comorbidities, such as prematurity, heart disease, and lung disease. However, it is worth noting that 60% of the total number of children in the study, including 65% of the children infected with RSV, had no comorbidities and were not eligible for the use of palivizumab, as well as half of the deaths occurred in children without comorbidities. In the largest RSV case series study, it was found that, although the fatality rate of the virus is higher in children with comorbidities, most cases of life-threatening RSV infection occur among previously healthy children from low- and middle-income countries, and the majority of children who died from RSV infection were younger than 6 months of age.^10^

Although only 2% of children with RSV infection require ICU admission for mechanical ventilation, it is estimated that ICU care represents 18% of total RSV-related hospital costs.^18^ The number of ICU admissions can be influenced by different ICU admission thresholds and an increasing variety of available respiratory support modalities. Therefore, providing resources based on scientific evidence and qualified care for children with severe cases of viral pneumonia at the entrance door to the health service can reduce ICU admissions that require mechanical ventilation, and thus, reduce the health system´s costs. Some studies demonstrated that there was a reduction in the length of hospital stay after implementing a change in medical practice to increase the use of non-invasive devices, such as the high-flow nasal catheter, which replaced invasive devices, specifically endotracheal tubes, central venous catheters and arterial catheters.^19^ In addition, a study carried out in Australia reported the suspension of low-value interventions, those not based on scientific evidence, such as chest radiography, salbutamol, corticosteroids, and epinephrine, which resulted in a decrease in the number of inappropriate treatments, but without changes in significant outcomes as length of stay or mortality.^20^

These data are relevant, once we are experiencing the pandemic of the new coronavirus, SARS-CoV-2, which has a lower number of severe cases and complications in children than observed in the adult population. Although more studies are still needed to determine its complications in the infant population, its current hospitalization and fatality rates are similar or lower than those of RSV infections in young children.^21–23^ Several studies showed a significant decrease in the number of children with RSV pneumonia in 2020 and 2021 worldwide, including Brazil.^24–26^ The most likely reason is that the use of personal protective equipment (PPE) introduced to address the COVID-19 epidemic has resulted in reduced transmission of other respiratory viruses. Nonetheless, the suspension of measures to combat the pandemic led to an increase in the number of other viral pneumonias in the Northern Hemisphere during the summer of 2021, out of seasonality.^27^ Thus, pediatric services should be alert to large outbreaks of RSV in the coming seasons, whose intensity may depend on the size of the epidemic during spring and summer at that location. Enhanced surveillance is recommended for both palivizumab administration and hospital capacity management.^28^

Regarding the different time intervals observed in the respiratory viruses surveillance system, the access to the hospital and to the ICU can be considered timely, in addition to a short median time of ICU stay, corroborating the acute nature of the condition. During a typical uncomplicated viral respiratory infection, the severity of symptoms increases around the second and third day of illness, and most symptoms gradually resolve between the seventh and tenth day, when complications do not develop. The median time of five days from the onset of symptoms to the collection of a nasopharyngeal sample for viral detection can be considered long, risking to generate false negative results, since PCR sensitivity is greater when collected within five days of the beginning of symptoms, period of greater viral shedding.^29^ Ideally, collection should be performed as soon as the patient is admitted to the hospital in order to reduce this time. Likewise, the period of viral detection results from sample collection was very long, as it does not allow for prompt diagnosis of viral infection and delays clinical decision-making consistent with the etiology of the patient’s infection. This can be observed by the high proportion of antibiotic prescriptions to treat these conditions of viral origin, which, in most cases, are unnecessary for their resolution. Therefore, the use of antibiotics can be considered inappropriate and can lead to the emergence of adverse events related to these medications, increased hospitalization time and costs, as well as consequences such as the induction of bacterial resistance to antibiotics in the hospital environment. One of the main cornerstones of antimicrobial stewardship programs to reduce bacterial resistance is to avoid the use of antibiotics to treat viral infections.^30^

Delay in results can also lead to inappropriate prescription of oseltamivir, an antiviral with exclusive action against the influenza virus. According to the Ministry of Health’s influenza treatment protocol,^7^ oseltamivir should be started in all SARS cases or in cases of ILI in patients with risk factors for influenza complications. However, this recommendation is based on studies conducted in the adult population, where influenza was the predominant virus among SARS cases, prior to the ­COVID-19 pandemic. Among children, the empirical use of oseltamivir for all SARS cases is controversial, since the main virus causing viral pneumonia is RSV and the clinical picture of influenza is indistinguishable from other respiratory viruses.^31^ In fact, our study corroborates this finding. Oseltamivir was prescribed to half of the patients, and only a small proportion had influenza virus infection. And likewise, a similar proportion of those who did not receive oseltamivir had influenza virus infection. Its use in children should be restricted to severe cases with timely laboratory diagnosis confirming influenza virus infection. Its empirical use could be justified only when epidemiological data show that it is an influenza epidemic period.

Our study has limitations. The acceptability of the surveillance system has gradually increased over the years, with a greater number of samples collected year after year; therefore, the initial years are less represented than the final years of the study. In addition, during the study period, rhinovirus was only incorporated into the viral panel in 2019, which may have caused unidentified cases, since rhinovirus is one of the main viruses responsible for viral pneumonia in children under five years of age.^2^ We did not collect data about the use of palivizumab, thus it was impossible to assess its effectiveness, since it is not possible to state whether the cases with comorbidities had received the medication properly. Finally, the study represents only one health unit, not allowing its results to be generalized to others, especially in a country as diverse as Brazil. It should be noted that this analysis ends in 2019, before the COVID-19 pandemic, because in the Federal District government’s response to the pandemic, the HMIB was initially selected as a backup pediatric hospital for cases of non-respiratory nature, while children with respiratory conditions suspected of COVID-19 were transferred to another referral hospital.

In conclusion, viral pneumonia has a great impact on pediatrics health services, even before the COVID-19 pandemic. These data only reinforce the importance of developing studies, carried out during the pandemic, regarding forms of pharmacological and non-pharmacological prevention, and clinical management of respiratory infections, not only caused by SARS-CoV-2, but also by others viruses. In addition, we point out the need for future studies regarding the effectiveness of oseltamivir use in children and the development of protocols aimed at the pediatric population, as well as the need of studies regarding the effectiveness of palivizumab in preventing RSV infections in high-risk groups and in those without comorbidities.

As for the surveillance system, it is essential to adopt strategies that reduce the time from hospitalization to sample collection and the time for the availability of results. Collection protocols and laboratory logistics should be improved, so that collection is done timely and PCR results are available within 24–48 hours, helping the etiological diagnosis and specific clinical treatment, and reducing the prolonged and unnecessary use of antibiotics. Moreover, the system should have sufficient capacity to quickly include emerging viruses, such as ­SARS-CoV-2, in the broad search for respiratory viruses, so that the surveillance system is not interrupted and remain useful in the early detection of emerging viruses infections; it would contribute to the response to public health emergencies, meeting the objectives that were set out to accomplish.^32^

## Data Availability

The database that originated the article is available with the corresponding author.
